# Prehabilitation for Patients with Brain Tumours: A Single-Centre Retrospective Cohort Study

**DOI:** 10.3390/curroncol33050242

**Published:** 2026-04-24

**Authors:** Kevin Y. Sun, Derek S. Tsang, Laura K. Langer, Alejandro S. Moreno, Amy E. Yeung, Alan K. H. Tam, Mark Bayley, Meiqi Guo

**Affiliations:** 1 Temerty Faculty of Medicine, University of Toronto, Toronto, ON M5S 1A1, Canada; 2 Radiation Medicine Program, Princess Margaret Cancer Centre, University Health Network, Toronto, ON M5G 2C4, Canada; derek.tsang@uhn.ca (D.S.T.); alejandro.shirimoreno3@uhn.ca (A.S.M.); 3 Toronto Rehabilitation Institute, University Health Network, Toronto, ON M5G 2C4, Canada; laura.langer@uhn.ca (L.K.L.); alan.tam@uhn.ca (A.K.H.T.); mark.bayley@uhn.ca (M.B.); meiqi.guo@uhn.ca (M.G.); 4 Queen’s Health Sciences, Queen’s University, Kingston, ON K7L 3N6, Canada; 23kdt1@queensu.ca; 5 Division of Physical Medicine and Rehabilitation, Department of Medicine, University of Toronto, Toronto, ON M5S 1A1, Canada

**Keywords:** brain neoplasms, prehabilitation, neurorehabilitation, functional outcomes, inpatient rehabilitation, acquired brain injury

## Abstract

Patients following brain tumour resection experience significant disability, yet rehabilitation is not typically delivered prior to adjuvant treatment such as radiation or chemotherapy. This study aims to characterize the medical and functional profiles, and function outcomes of patients with brain tumour admitted over the past four years to a pilot inpatient prehabilitation programme following brain tumour resection but prior to adjuvant therapy, and to compare these findings with those of patients in a standard acquired brain injury rehabilitation programme. We retrospectively reviewed the charts from a randomly selected sample of 58 prehabilitation inpatients and 112 patients with acquired brain injuries at Toronto Rehabilitation Institute between March 2020 and December 2024. Data abstracted included demographics, medical and functional profiles, Functional Independence Measure (FIM) scores, and discharge parameters. Compared with acquired brain injury subjects, prehabilitation subjects had significantly less physical (47% vs. 86%, *p* < 0.0001) but more communication (46% vs. 20%, *p* = 0.0005) impairments, though with similar mean FIM change (22.5 vs. 26.0, *p* = 0.082) and FIM efficiency (1.1 vs. 1.0, *p* = 0.78). While not reaching significance, they also experienced more mood issues during rehabilitation (30% vs. 18%, *p* = 0.075). These findings support that prehabilitation after brain tumour surgery but before adjuvant therapy is clinically effective within existing ABI rehabilitation programmes. However, prehabilitation programmes may benefit from staffing models that emphasize communication supports and mental health expertise.

## Simple Summary

Patients after brain tumour surgery often experience difficulties with thinking, movement and communication, yet rehabilitation is not typically delivered prior to adjuvant treatment. This study examined outcomes from a novel prehabilitation programme delivered after surgery but before radiation and/or chemotherapy. We reviewed the records of 58 patients with brain tumours who received prehabilitation and compared them to 112 patients in a standard brain injury rehabilitation programme (patients without brain tumours). We found that although patients with brain tumours showed smaller improvements in function, their recovery rate was similar to the comparison group. They also were more likely to have communication impairments and mood difficulties but were less likely to have physical impairments. These results suggest that prehabilitation may be helpful for improving function in patients with brain tumours. However, such programmes may require additional institutional staff resources to better support patients’ communication and mental health needs.

## 1. Introduction

Primary brain tumours present a significant clinical challenge for patients due to tumour-related impairments, surgical complications, and radiation-induced neurotoxicity that can significantly diminish quality of life [1]. In Canada, approximately 3300 individuals are diagnosed with brain and spinal cord tumours annually, and an estimated 2600 die from the disease, representing an age-standardized mortality rate of 6.7 per 100,000 [2,3]. Although survival has improved with advances in neurosurgical and oncologic care [4], many patients experience significant functional decline following tumour resection and during adjuvant therapy, highlighting the need for targeted rehabilitation strategies [5,6].

Inpatient prehabilitation programmes aim to optimize a patient’s physical, cognitive, and psychological readiness for the physiological stress associated with acute treatments, such as surgery or adjuvant therapies, including chemotherapy or radiation therapy [7]. Evidence from a large network meta-analysis demonstrated that prehabilitation significantly reduces postoperative complications and enhances quality of life across surgical populations [8]. However, in the context of primary brain tumours, rehabilitation prior to surgery is often not feasible, as neurosurgical procedures are frequently performed on an urgent basis following diagnosis. In this study, we use the term prehabilitation to refer to rehabilitation delivered after tumour resection to optimize function prior to the initiation of adjuvant oncologic therapy. Consistent with this concept, a randomized control trial has shown that structured cognitive training delivered during this interval improved cognitive outcomes, particularly visual attention and verbal memory [9].

Inpatient rehabilitation has been shown to improve functional outcomes and quality of life in patients with brain tumours across a range of disease stages and treatment trajectories. However, existing evidence has primarily examined rehabilitation delivered during or following adjuvant oncologic therapies [10,11,12,13]. These studies demonstrate that patients with brain tumours receiving rehabilitation during or after adjuvant therapy have similar FIM efficiency to that observed in established acquired brain injury rehabilitation programmes [10,11,12]. Consequently, there is limited understanding of the role of structured inpatient prehabilitation, delivered post-operatively before the influence of further oncologic treatment. This interval represents a unique opportunity for rehabilitation: rather than passively awaiting post-operative pathology results and arrangements for adjuvant therapy, patients may instead engage in goal-directed therapies to optimize functional recovery prior to the treatment-related effects of radiation and/or chemotherapy. In response to this gap, a pilot inpatient brain tumour prehabilitation programme was initiated at the Toronto Rehabilitation Institute (TRI) in 2020 to support patients during this peri-treatment interval. This knowledge gap is particularly relevant in the Canadian publicly funded healthcare system, where evidence guiding the timing and structure of inpatient rehabilitation for patients with brain tumours remains limited, restricting public funding for this service.

The primary objective of this study was to characterize the medical and functional profiles, as well as functional outcomes of patients admitted over four years to a prehabilitation pilot inpatient programme following brain tumour resection but prior to radiation or chemotherapy. The secondary objective was to compare these findings with those of patients in a standard acquired brain injury rehabilitation programme serving as an established inpatient neurorehabilitation reference group. By combining domain-specific functional assessments with standardized rehabilitation outcomes and survival analyses, this study provides a descriptive framework for understanding patient characteristics and outcomes for an inpatient brain tumour prehabilitation programme.

## 2. Materials and Methods

### 2.1. Study Design and Population

This single-centre retrospective cohort study was conducted in the acquired brain injury rehabilitation programme (ABI) at the Toronto Rehabilitation Institute (TRI), an academic rehabilitation hospital in Toronto, Ontario, Canada. There is a total of 33 beds dedicated to patients with acquired brain injury, and the average length of stay is about 30 days. The ABI rehab programme traditionally served patients with traumatic brain injury, encephalitis, toxic/metabolic brain injuries, anoxic brain injuries, and grade 1 meningiomas (not requiring radiation or chemotherapy immediately after surgery). However, in March 2020, the programme also started to accept patients with gliomas and brain metastases for inpatient prehabilitation to better align with local patient population needs.

The neurorehabilitation programme at TRI follows an interdisciplinary model aimed at addressing the physical, cognitive, and communication impairments involved in acquired brain injury. Patients typically receive coordinated care from a team including physiatrists, physiotherapists, occupational therapists, speech–language pathologists, neuropsychologists, dietitians, nurses, and social workers. Rehabilitation interventions may include mobility and strength training, activities of daily living retraining, cognitive rehabilitation, communication and swallowing therapy, as well as psychosocial support. The duration of the rehabilitation programme was individualized based on patient-specific functional needs, with the goal of the patients being discharged prior to the start of their adjuvant treatments.

The study period spanned from 11 March 2020 to 31 December 2024. Eligible participants included adult patients admitted to the acquired brain injury (ABI) inpatient rehabilitation programme at the Toronto Rehabilitation Institute during this period. Inclusion criteria consisted of patients admitted with either a brain tumour diagnosis requiring prehabilitation or other acquired brain injury diagnoses managed within the programme. The prehabilitation group included patients with gliomas, brain metastases, grade 2 or 3 meningiomas, and other diagnoses with planned postoperative adjuvant therapy. The general ABI group included patients admitted for other acquired brain injuries such as traumatic brain injury, encephalitis, toxic or metabolic brain injury, anoxic brain injury, and grade 1 meningiomas not requiring immediate adjuvant oncologic treatment.

Based on estimated annual admissions of 50–70 brain tumour patients receiving prehabilitation and 240–350 other ABI patients (total population range 1415–1680), a required sample size of 168 charts was calculated to achieve a 90% confidence level, 5% precision, ≤5% missing data, and accommodate 12 variables of interest. Sample size calculations were performed in SAS 9.4 (SAS Institute, Cary, NC, USA).

From the eligible admissions, 58 patients with brain tumours (prehabilitation group) and 112 patients with other ABI diagnoses (general ABI group) were randomly selected in a 2:1 ratio to reflect underlying admission patterns while maintaining statistical power. Random selection was performed using the random generator function in Microsoft Excel applied to eligible cases on a programme admission list.

Clinical data, which included medical co-morbidities, education level, the presence of physical, cognition, communication and/or behavioural impairments, the presence of mood issues, readmissions to acute care, and participation in outpatient rehabilitation after inpatient discharge, was extracted from discharge summaries, consults, and progress notes in the electronic medical records and from public obituary records (when applicable) using a standardized abstraction form. The presence of physical, cognition, communication and/or behavioural impairments, and the presence of mood issues were determined based on the treating physiatrists’ documented clinical assessment in their consult notes. Additional data was also gathered from the Decision Support department at the University Health Network (UHN). Data abstracted included the following demographic and clinical attributes:

- Age;
- Gender;
- Level of education;
- Past medical history;
- Type of impairments;
- Length of stay (LOS);
- Admission and discharge Functional Independence Measure (FIM);
- Discharge to outpatient rehabilitation;
- Service interruptions during inpatient rehabilitation (temporary transfers from inpatient rehabilitation to acute care due to intercurrent medical complications).

The Functional Independence Measure (FIM) is an 18-item assessment tool that measures the degree of disability and functional independence in rehabilitation patients across two primary domains: motor function (13 items) and cognitive function (5 items). Each item is scored from 1 to 7, with higher scores representing greater independence, ranging from complete dependence to complete independence [14]. Charlson Comorbidity Index, a weighted index used for examining comorbidities to predict mortality, was used to quantify the past medical history of subjects [15]. FIM change was calculated by subtracting the admission FIM from the discharge FIM, and a positive FIM change indicates functional gains. FIM efficiency was calculated as FIM change divided by LOS, representing the rate of functional change per day of rehabilitation.

In addition, for subjects in the prehab group, the following oncological information was abstracted:

- Tumour grade;
- Tumour type;
- Tumour laterality;
- Adjuvant treatments;
- Survival.

### 2.2. Statistical Analysis

Descriptive statistics were used to summarize the demographic and clinical data. Counts and percentages were used for categorical variables and mean (standard deviation) for normally distributed continuous variables and median (interquartile range, IQRR) for not normally distributed continuous variables. Group differences in baseline covariates for prehabilitation and other ABI groups were assessed using t-tests and chi squares. Kaplan–Meier was used to determine median time to event (disease related mortality) with participants lost to follow-up right censored in prehab stream. An ANCOVA was used to determine group differences in FIM change and FIM efficiency, adjusted for age and admission FIM. A logistic regression was performed to analyze discharge to outpatient rehab by stream. A *p* value < 0.05 will be considered statistically significant. All analyses were completed using SAS 9.4 (SAS Institute, Cary, NC, USA).

### 2.3. Ethics Approval

The University Health Network Research Ethics Board approved this study (CAPCR #25-5282; date of approval: 15 April 2025). Due to the retrospective design involving the review of existing medical records, the requirement for informed consent was waived by the Research Ethics Board. Many patients were deceased or lost to follow-up at the time of data collection, making direct consent impracticable. The study was considered minimal risk, and all identifiable health information was handled in accordance with institutional privacy policies with restricted access to authorized study personnel.

## 3. Results

### 3.1. Demographics

A total of 170 patients were included in the study, comprising 112 patients in the ABI stream and 58 in the prehabilitation stream. In the prehabilitation stream, three patients were unable to complete inpatient rehabilitation due to acute medical issues during rehabilitation requiring transfers back to acute care. As a result, complete sets of FIM scores were not available for these patients. In the general ABI stream, one patient did not complete rehabilitation for the same reason.

Demographic and medical characteristics of the subjects are reported in Table 1. Individuals in the prehabilitation stream were younger than those in the general ABI stream (mean [SD] age, 49.9 [16.4] vs. 57.4 [20.7], *p* = 0.019), had higher Charlson Comorbidity Index scores (3.4 [1.2] vs. 2.2 [2.2], *p* < 0.0001), and were less likely to be discharged to outpatient rehab than ABI patients (39% vs. 86%, *p* = 0.0003). There were no significant differences between groups in sex distribution (44% vs. 38%, *p* = 0.45).

Compared with patients in the ABI stream, patients receiving prehabilitation demonstrated a distinct impairment profile at admission. Patients in the prehabilitation stream were significantly less likely to exhibit physical impairments (47% vs. 86%, *p* < 0.0001) but were more likely to have communication impairments (46% vs. 20%, *p* = 0.0005). The prevalence of cognitive impairments was high in both groups and did not differ significantly (75% vs. 86%, *p* = 0.10), nor did rates of behavioural issues (14% vs. 10%, *p* = 0.45). Mood issues were more frequently observed in the prehabilitation cohort compared with ABI patients, although this difference did not reach statistical significance (30% vs. 18%, *p* = 0.075).

Patients in the prehabilitation stream had a lower odds ratio of attending outpatient rehabilitation after inpatient rehabilitation than ABI (OR 0.30, 95% CI 0.15–0.58, *p* = 0.0004). Only 39% later attended outpatient rehabilitation compared to 86% of ABI patients. Prehabilitation patients also had higher rates of readmission to acute care than ABI patients when adjusted for length of stay (14% vs. 5%, *p* = 0.033).

Tumour characteristics and treatment details of the prehabilitation stream are reported in Table 2. Among prehabilitation patients, the most common brain tumour type was glioma (87%), followed by brain metastases (7%). Furthermore, brain tumours were more commonly lateralized to the left (58%). Most prehabilitation patients did go on to receive chemotherapy (81%) and/or radiation therapy (86%) following prehabilitation.

**Figure 1:**
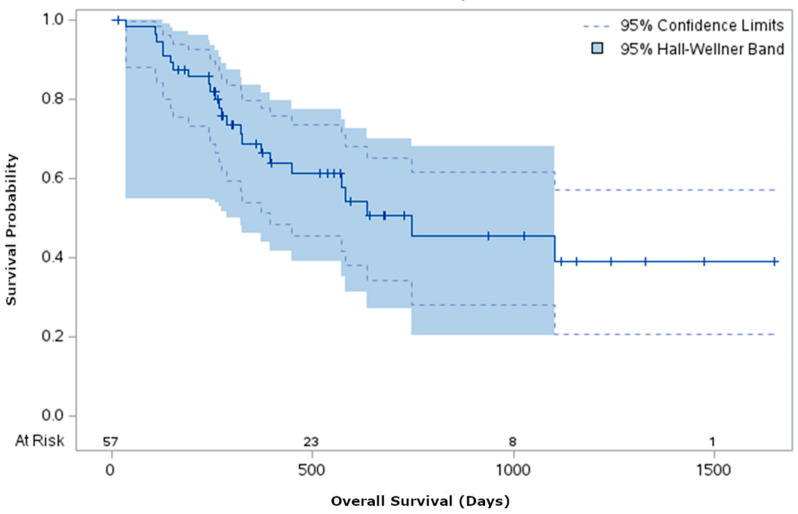
Overall survival time of patients in the prehabilitation group from date of admission to all-cause mortality was descriptively estimated using a Kaplan–Meier curve. Median survival was 748 days (95% CI 393 days to non-estimable upper value). Shaded areas represent 95% confidence bands estimated using the Hall–Wellner method. The final plateau reflects a single participant who remained event-free and was censored at their last follow-up. One subject was excluded because time-to-event could not be reliably determined due to discrepancies in recorded dates.

Survival analysis from the date of admission to event was conducted in the prehabilitation cohort for descriptive purposes to estimate overall survival (Figure 1). Median overall survival was 748 days (95% CI 393 days to non-estimable upper value). Confidence bands were estimated using the Hall–Wellner method to visualize the uncertainty around the survival curve.

### 3.2. Functional Outcomes

Prehabilitation patients had lower FIM change (22.5 vs. 26, *p* = 0.082) and shorter mean length of stay (24.6 [14.4] vs. 30.3 [14.9], *p* = 0.018) when compared with ABI patients (Table 3). There was no significant difference in FIM efficiency (1.1 vs. 1.0, *p* = 0.78), demonstrating similar benefits to rehabilitation regardless of length of stay and underlying diagnosis.

## 4. Discussion

This study characterized the demographic, medical, and functional profiles of patients with brain tumours receiving inpatient prehabilitation at an academic rehabilitation hospital following tumour resection but prior to the initiation of adjuvant therapy. We found that patients in the prehabilitation stream were significantly less likely to have physical impairments but were more likely to have communication impairments and mood issues during inpatient rehabilitation. Compared with the ABI cohort, prehabilitation patients achieved smaller absolute functional gains during shorter lengths of stay in rehabilitation but demonstrated similar rates of functional improvement as measured by FIM efficiency. In other words, prehabilitation patients with brain tumours seem to benefit similarly to a general ABI population from inpatient ABI rehabilitation. Moreover, this population with brain tumours experiences a median survival of over 2 years, demonstrating a potential for longitudinal functional benefits with a short prehabilitation intervention of less than 30 days duration, even among individuals with an oncologic diagnosis.

These findings have several implications for how rehabilitation programmes for these patients with brain tumours may be designed for optimal delivery in the future. First, patients admitted to the prehabilitation stream demonstrated a distinct admission impairment profile, characterized by relatively preserved physical function but higher rates of communication and mood-related difficulties. The lower physical impairment likely reflects both the more focal nature of tumour-related deficits as compared to diffuse traumatic brain injuries and the selection of patients sufficiently well to participate in prehabilitation. In contrast, communication impairments were more prevalent in the prehabilitation cohort. This may be influenced by tumour location, as language and higher-order communication functions are mainly left-lateralized. Gliomas, which made up most of the tumours in our cohort, typically involve the frontal and temporal lobes, which are regions essential for speech and language, providing a plausible anatomical explanation for the communication deficits [16]. Mood difficulties were also more common, reflecting the well-known impact of brain tumours on emotional well-being. Depression and personality changes often arise in this context, driven by the stress of the diagnosis, uncertainty about functional abilities, and the anticipation of cancer treatments [17,18]. Palliative care guidelines from the European Association for Neuro-Oncology similarly emphasize the importance of routine screening and integrated psycho-oncological care in this population [19]. Collectively, these findings underscore the need for prehabilitation models that integrate targeted communication interventions and mental health expertise to address the complex functional and psychosocial needs of patients recovering from brain tumour surgery.

Second, our analyses of FIM efficiency, FIM change, and length of stay are consistent with prior studies in neuro-oncology rehabilitation. O’Dell et al. [11] and Huang et al. [12] similarly demonstrated comparable daily functional gains between patients with brain tumours and those with traumatic brain injury, despite shorter rehabilitation lengths of stay and smaller absolute functional improvements in the brain tumour cohorts [11,12]. In the present study, the shorter length of stay observed in the prehabilitation group is likely attributable to constraints imposed by planned adjuvant therapies and the goal of optimizing functional status to enable discharge home following inpatient rehabilitation. In addition, FIM efficiency was comparable between the prehabilitation and the ABI rehabilitation groups, and the absolute FIM change in this study’s prehabilitation group was also similar to FIM changes published in the literature for the neuro-rehabilitation inpatient population [20,21]. Together, these findings demonstrate that patients with brain tumours derive functional benefit from inpatient prehabilitation comparable to ABI populations that have historically been admitted to inpatient ABI rehabilitation.

In addition, the results also demonstrated that patients in the prehabilitation cohort experienced more frequent interruptions to rehabilitation due to medical issues that required transfer back to acute care compared with the general ABI population. This aligns with the previous literature showing that patients with brain tumours face a higher risk of acute hospital transfer during inpatient rehabilitation, with reported rates of approximately 20.2% versus 9% for patients undergoing rehabilitation for traumatic brain injury [22,23]. These transfers are often related to neurologic complications, treatment effects, or disease progression, highlighting the importance of close medical monitoring by rehabilitation physicians for this patient population during inpatient rehabilitation [23], as well as close communication with neurosurgical and oncology care teams.

Lastly, patients undergoing inpatient prehabilitation in the study demonstrated a median survival of approximately two years, suggesting that rehabilitation for this population may extend beyond purely palliative aims. At the same time, a much lower percentage of patients in the prehabilitation group (39%) in this study attended outpatient therapy after discharge from inpatient rehabilitation than the ABI rehabilitation group (86%). While this difference may reflect the burdens of adjuvant chemotherapy or radiation, tumour progression, or post-treatment complications for patients in the prehabilitation group, prior survivorship studies have demonstrated that a subset of brain tumour survivors, particularly younger patients with preserved functional status and lower disease burden, are able to return to work or other complex life roles following treatment. For example, Basalathullah et al. [24] reported that over 60% of survivors of primary brain tumours treated with radiotherapy returned to part-time or full-time employment, while Nieder et al. [25] similarly demonstrated sustained employment among select long-term survivors of brain metastases [24,25]. Taken together with the observed survival in our cohort, these findings suggest not only the clinical value of structured inpatient rehabilitation but also the need for outpatient rehabilitation planning for select patients with brain tumours to pursue meaningful higher-level functional goals during survivorship.

This study should be interpreted in the context of several limitations. The single-centre design may limit generalizability given international variability in rehabilitation delivery and funding models. In addition, the average age of the prehabilitation cohort in this study was younger than the average age of patients with brain tumours described in the literature [26], likely reflecting selective referral and limiting applicability to older patients with brain tumours. Although the tumour types in this study reflected known epidemiologic patterns, lesions were disproportionately left-sided, whereas population-based data typically show an even distribution between hemispheres [27]. However, left-hemispheric tumours often produce more functionally visible deficits, such as aphasia, which may have contributed to the decision for referral for inpatient rehabilitation. Survival analyses for the prehabilitation cohort were also constrained by exclusion of patients with missing or invalid time-to-event data, small subgroup sizes, sparse events, and high censoring, which reduced statistical power and precision; accordingly, null findings should be interpreted cautiously. In addition, the study period began during the COVID-19 pandemic, when disruptions to hospital operations may have affected rehabilitation delivery. Furthermore, there is heterogeneity in diagnostic composition between the prehabilitation and ABI comparator groups, which may influence rehabilitation outcomes and limit interpretation of between-group comparisons. However, comparisons with broader acquired brain injury populations are commonly used in neurorehabilitation research as a pragmatic reference group, and the randomly sampled ABI cohort in this study reflects the typical case mix encountered in routine clinical practice [11,12]. Finally, as a retrospective study, analyses were limited to routinely collected clinical data, with functional outcomes measured exclusively using the Functional Independence Measure. While widely used, the FIM has limited sensitivity to cognitive change, is subject to ceiling effects, and does not capture patient-reported outcomes such as quality of life [28].

Despite these limitations, this study has several strengths. This is a cohort of patients that spans across four years and reflects typical clinical referrals and treatment in routine inpatient rehabilitation, which enhances the real-world applicability of our findings. Unlike many studies with strict inclusion criteria, this cohort captures the heterogeneity of patients seen in practice, including differences in age, impairment profiles, and comorbidities. We were also able to compare these patients to the broader population of individuals with acquired brain injuries, providing context for functional outcomes. Finally, detailed chart reviews allowed us to capture clinical characteristics not typically available in administrative datasets, including the prevalence of mood issues and specific types of cognitive, motor, and functional impairments.

In conclusion, patients undergoing brain tumour resection represent a distinct rehabilitation population and could be considered for inpatient prehabilitation to optimize functional recovery prior to adjuvant therapy. Although patients in the prehabilitation stream achieved smaller absolute functional gains, their rate of functional improvement per rehabilitation day was comparable to that observed in established ABI populations, supporting the clinical effectiveness of prehabilitation within existing ABI rehabilitation programmes. The higher prevalence of communication and mood-related impairments highlights the importance of individualized, multidisciplinary rehabilitation models incorporating enhanced psycho-oncological and communication-focused supports. Prospective studies with larger cohorts and more detailed oncologic treatment data are needed to examine the relationships among rehabilitation and adjuvant therapy, and to inform optimal prehabilitation programme planning for patients with brain tumours.

## Figures and Tables

**Table 1:** Demographics of prehabilitation patients and acquired brain injury patients.

	Prehabilitation (*n* = 58)	ABI (*n* = 112)	*p*
Female	25 (44%)	42 (38%)	0.45
Age (SD)	49.9 (16.4)	57.4 (20.7)	0.019
Years of Education (SD)	16.3 (2.8)	15.3 (4.1)	0.09
Admission FIM (SD)	76.8 (21.8)	80.4 (21.5)	0.39
Charleston Comorbidity (SD)	3.4 (1.2)	2.2 (2.2)	<0.0001
Attended Outpatient Rehabilitation after Inpatient Rehabilitation	22 (39%)	76 (86%)	0.0003
Readmission to Acute Care	14%	5%	0.033
Physical Issues	27 (47%)	96 (86%)	<0.0001
Behavioural Issues	8 (14%)	11 (10%)	0.45
Cognitive Issues	43 (75%)	96 (86%)	0.10
Mood Issues	17 (30%)	20 (18%)	0.075
Communication Issues	26 (46%)	22 (20%)	0.0005

**Table 2:** Tumour characteristics and treatment details of prehabilitation patients.

Characteristic	Prehabilitation (*n* = 58)
Tumour Grade	
1	2 (4%)
2	9 (17%)
3	8 (15%)
4	35 (65%)
Tumour Type	
Glioma	47 (87%)
Brain Metastasis	4 (7%)
Meningioma	1 (2%)
Other	5 (9%)
Laterality	
Left	33 (58%)
Right	20 (35%)
Bilateral/Midline	4 (7%)
Chemotherapy	46 (81%)
Radiation Therapy	49 (86%)
Median Survival (days)	748

**Table 3:** Functional outcomes in patients undergoing prehabilitation compared with acquired brain injury patients.

Outcome	Prehabilitation (*n* = 58)	ABI (*n* = 112)	*p*
∆FIM (IQR)	22.5 (14–34.5)	26 (17–41)	0.082
Admission FIM (IQR)	82.5 (68–95)	80.5 (63.5–100.5)	0.82
Discharge FIM (IQR)	111.5 (102.5–115.5)	114 (105–119)	0.069
FIM Efficiency (IQR)	1.1 (0.65–1.49)	1.0 (0.62–1.50)	0.78
Length of Stay (days, SD)	24.6 (14.4)	30.3 (14.9)	0.018
Admission FIM-M (SD)	59.5 (17.9)	58.6 (20.5)	0.71
Discharge FIM-M (SD)	77.7 (18.6)	81.3 (13.9)	0.21
Admission FIM-C (IQR)	22 (18–24)	21 (18–24.5)	0.83
Discharge FIM-C (IQR)	26.5 (23.5–29)	28.0 (24–31)	0.11
∆FIM-M (IQR)	18 (9.5–28)	21 (11–33)	0.13
∆FIM-C (IQR)	5 (2–7)	6 (3–9)	0.075

ΔFIM refers to change in FIM scores from admission to discharge; FIM-M refers to the motor subscale and FIM-C refers to the cognitive subscale of the FIM; and IQR refers to the interquartile range.

## Data Availability

The data presented in this study are available on request from the corresponding author. The data are not publicly available due to privacy and ethical reasons.

## Abbreviations

FIM	Functional Independence Measure
LOS	Length of Stay
ABI	Acquired Brain Injury
TRI	Toronto Rehabilitation Institute
UHN	University Health Network
